# Supplementary material for: The interactome of intact mitochondria by cross-linking mass spectrometry provides evidence for coexisting respiratory supercomplexes
Source: Mol Cell Proteomics. 2017 Dec 8;17(2):216–32. doi: 10.1074/mcp.RA117.000470 (PMC5795388; doi:10.1074/mcp.RA117.000470)
Supplement: Supplemental Data [file supp_RA117.000470_133922_0_supp_23978_fzffwf.zip › spectra_annotation/mito_spectra_annotation/196-1-6-1-4-1.pdf]

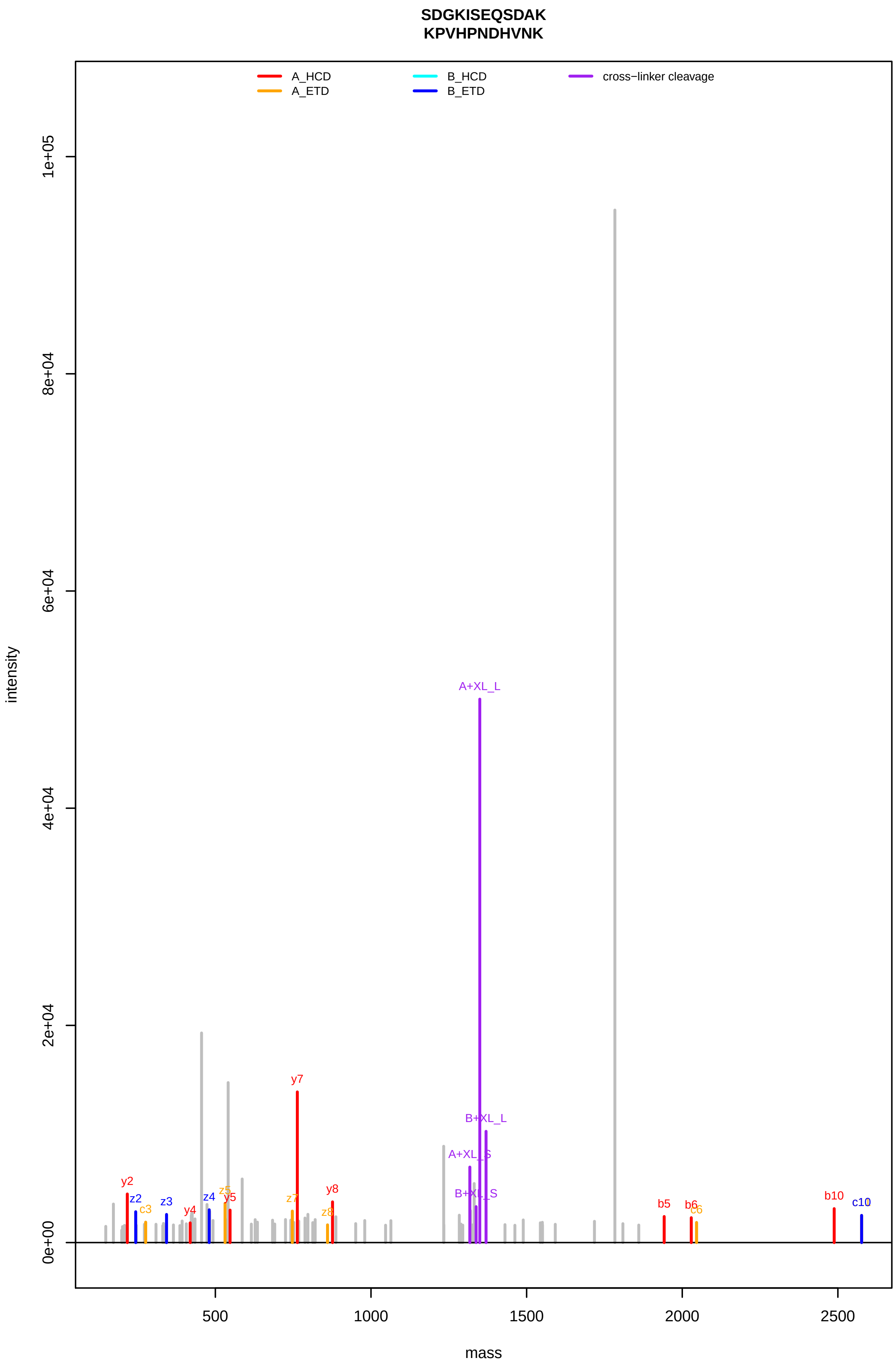
SDGKISEQSDAK
KPVHPNDHVNK
A_HCD
A_ETD
B_HCD
B_ETD
cross−linker cleavage
intensity
mass
A+XL_L
B+XL_L
A+XL_S
B+XL_S
y7
y2
z2
c3
z3
y4
z4
z5
y5
z7
y8
z8
b5
b6
c6
b10
c10

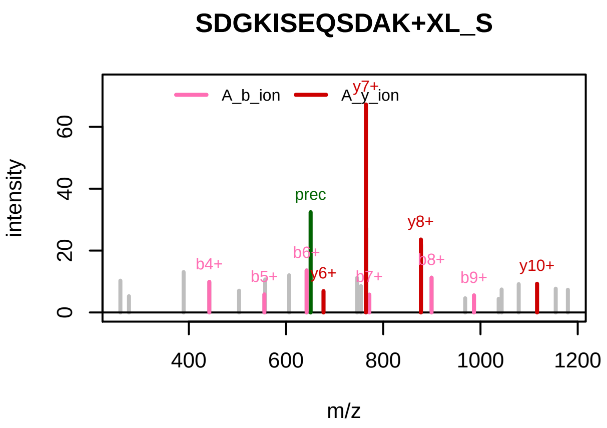
SDGKISEQSDAK+XL_S
A_b_ion
A_y_ion
intensity
m/z
prec
y7+
y8+
y10+

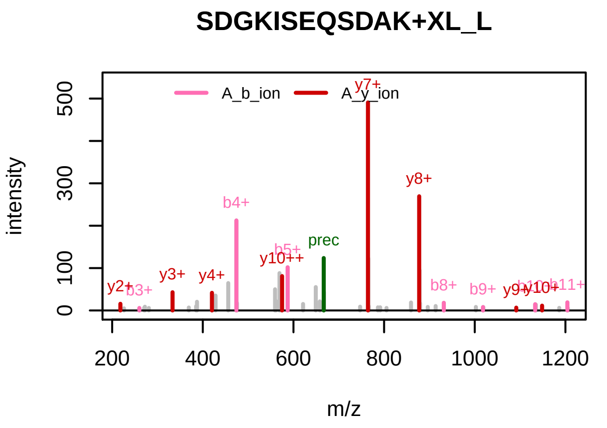
SDGKISEQSDAK+XL_L
A_b_ion
A_y_ion
intensity
m/z
y7+
y8+
b4+
prec
y3+
y4+
y2+

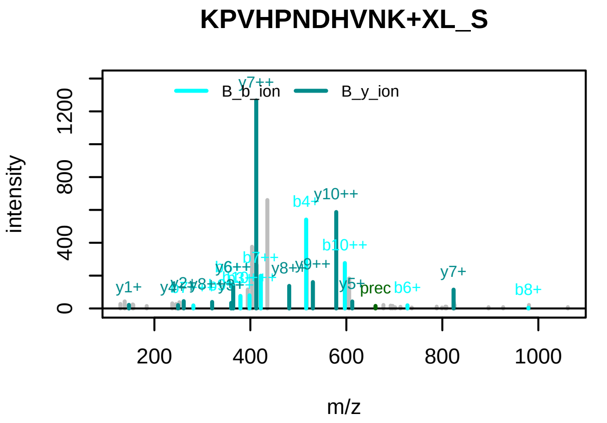
KPVHPNDHVNK+XL_S
B_b_ion
B_y_ion
intensity
m/z
y10++
b4+
b10++
y7+
b8+
y1+
prec
b6+

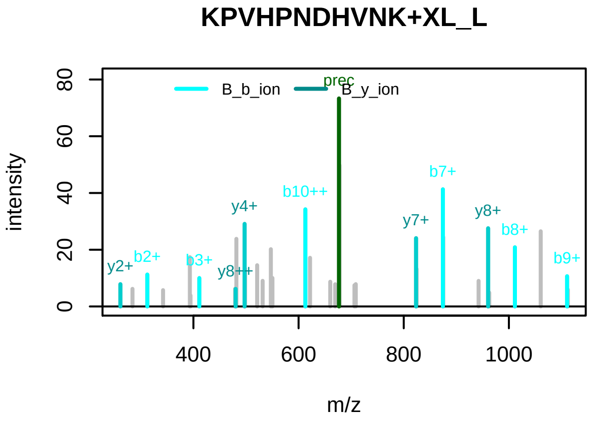
KPVHPNDHVNK+XL_L
B_b_ion
B_y_ion
intensity
m/z
prec
b7+
b10++
y4+
y7+
y8+
b8+
b9+
y2+
b2+
b3+
y8++
